# Negative affect and respiratory sinus arrhythmia are differentially related to social anxiety and autism features in autistic preschoolers contrasted to fragile X syndrome

**DOI:** 10.3389/fpsyt.2023.1151263

**Published:** 2023-03-20

**Authors:** Carla A. Wall, Jane E. Roberts

**Affiliations:** ^1^Duke Center for Autism and Brain Development, Duke University Medical Center, Durham, NC, United States; ^2^Department of Psychology, University of South Carolina, Columbia, SC, United States

**Keywords:** autism, fragile X syndrome, social anxiety, temperament, respiratory sinus arrhythmia

## Abstract

**Introduction:**

Autism spectrum disorder (ASD) is a highly heterogeneous and complex disorder with co-occurring disorders commonplace. This presents tremendous diagnostic challenges given the phenotypic overlap between autism and other diagnoses, including social anxiety, as well as variance in specific genetic disorders like fragile X syndrome (FXS). Biobehavioral measurement approaches integrate behavioral and biological data, and by so doing have the potential to address diagnostic challenges and shed light on the mechanisms underlying social impairments.

**Methods:**

The present study utilized a biobehavioral approach to evaluate how biologically based indices of baseline respiratory sinus arrhythmia (RSA) and temperamental negative affect differ and predict autism and anxiety in a sample of 120 preschoolers with non-syndromic autism (nsASD) with co-occurring intellectual impairment, FXS, and neurotypical (NT) development.

**Results:**

Results indicated that children with nsASD display elevated negative affect compared to both FXS and NT controls which did not differ from each other and females exhibited more negative affect relative to males. Interestingly, elevated negative affect predicted social anxiety, but not ASD in FXS. Baseline RSA did not differ across the groups; however, reduced RSA predicted elevated autism severity for the nsASD group but not those with FXS or NT development.

**Discussion:**

Taken together, biobehavioral markers differentiated the groups in discrete ways that advance our understanding of autism and promote improved diagnostic clarity using objective measurement.

## Introduction

Autism^1^ is a highly prevalent neurodevelopmental condition characterized by difficulties with social communication and the presence of restricted and repetitive interests and behaviors (5, 6). Social anxiety is a specific category of anxiety that, much like autism, is associated with social symptoms including avoidance, anxious anticipation, or distress in social situations (5). Because both autism and social anxiety have significant overlap in behavioral symptoms and likely have genetic underpinnings, differentiating between when these disorders occur independently or jointly poses challenges (7). These challenges are magnified in young children with limited language or insight, which makes self-report measures inappropriate (8, 9). Despite these barriers, differential diagnoses of autism and social anxiety are critical to initiate targeted treatments known to reduce the impact of these diagnoses on overall quality of life (10, 11).

A biobehavioral measurement approach can help address diagnostic challenges by acknowledging that social behaviors (such as autism and anxiety features) are driven in part by biological underpinnings and utilizing multiple sources of information. Integrating a biobehavioral framework with a genetic model of autism has further potential to accelerate discoveries regarding the underpinnings of autism and its association with social anxiety. Both autism and social anxiety have clear genetic underpinnings, albeit with complex and mixed etiology, and their high co-occurrence suggests shared genetic influences (12, 13). There is a benefit to investigating the co-occurrence of multiple disorders within an identified genetic syndrome (14), and one of the most promising genetic models of autism is fragile X syndrome (FXS) (15, 16). FXS is a rare monogenic disorder caused by mutations on the *FMR1* gene and the ensuing under-expression of its associated fragile X messenger ribonucleoprotein [FMRP; (17)]. FXS is associated with a range of social impairments that are highly associated with both autism and social anxiety, including social avoidance and reduced eye contact (18, 19) as well as mild-to-moderate intellectual disability (20). Furthermore, FXS has the highest penetrance for any single gene disorder associated with autism; 50–75% of males and 25% of females with FXS meet diagnostic criteria for autism (21, 22). Individuals with FXS also have a high risk for anxiety, with recent studies finding that up to 62% of males and 100% of females with FXS meet DSM-5 criteria for social anxiety (16).

Temperamental negative affect refers to a biological tendency toward negative emotions such as fear, anger, or sadness (23). Negative affect is present in infancy and persists through toddlerhood, and has been linked to neural functioning, especially amygdala activation, and autonomic and behavioral responses (23). Negative affect is reliably measured using parent report (24), making it a useful and feasibly deployed biobehavioral marker in young children with and without neurodevelopmental disorders. Most research has focused on negative affect in neurotypical (NT) children with increased application to clinical groups observed recently (25–27). For example, Macari et al. (25) found that 26-month-old toddlers with non-syndromic autism (nsASD) have higher overall negative affect than their NT peers; this elevation was unique to nsASD and not observed in a matched group of developmentally delayed toddlers without nsASD. Using a developmental approach, studies of negative affect have shown that children with FXS demonstrate an accelerated increase in negative affect over time as compared to their NT peers (27, 28), despite cross-sectional studies showing that children with FXS have lower negative affect at certain ages (29). No study to date has directly compared negative affect in children with nsASD contrasted to FXS, leaving an important gap in our understanding of the role negative affect plays in the differential characterization of these populations.

Physiological regulation is another useful biobehavioral marker for differentiating social symptoms, as there is clear evidence that physiological regulation contributes to both cognitive and social competency (30, 31). Physiological regulation can be reflected through autonomic nervous system (ANS) activity, including both the sympathetic and parasympathetic nervous systems, and it can be easily measured non-invasively through cardiac indices. One such index is respiratory sinus arrhythmia (RSA), which assesses parasympathetic nervous system function by measuring the beat-to-beat variability in heart rate associated with respiration. The parasympathetic nervous system functions to slow heart rate and lower blood pressure to allow resources necessary to promote behavioral regulation (32), and RSA plays an important role in the physiological regulation of stress and social engagement (33, 34). For example, adaptive RSA suppression has been associated with fewer childhood internalizing and externalizing problems and greater emotion regulation (35).

Baseline RSA, or RSA measured during a neutral, unaroused state, is thought to be a measure of individual differences in arousal (35), and it has been used to understand underlying differences in physiological regulation in individuals with nsASD, FXS, and NT development (36). Studies have shown conflicting evidence for atypical RSA in nsASD, with some studies finding no evidence for differences between those with nsASD and those without [e.g., (37)], and some studies finding reduced RSA in nsASD (38). Longitudinal work has shown that infants who go on to develop nsASD show a smaller increase in RSA over time relative to their NT peers, suggesting that there may be developmental processes at play (39). Taken together, this evidence points to the need for additional research. Limited work has compared RSA in nsASD and other neurodevelopmental disorders including FXS, but studies with older children suggest that RSA does not differ between individuals with nsASD and FXS (37, 40). By contrast, a large body of work has documented maladaptive (i.e., lower) baseline RSA values in FXS compared to NT development from infancy (41, 42) through adolescence (43). Notably, no studies have included pre-school females with FXS, leaving critical gaps in our understanding of physiological regulation in this population.

In addition to providing descriptive information about underlying differences in genetic risk groups, biobehavioral measures have predictive utility in determining the presence and severity of disorders like autism and social anxiety. Many studies have investigated the role of negative affect as a useful predictor of later autism diagnosis in children with nsASD (44–46). In longitudinal prospective studies of infant siblings of children with nsASD, higher parent-reported negative affect in infancy has been associated with autism outcomes in toddlerhood in multiple independent studies (44–46). Importantly, the association of higher negative affect to increased autism diagnoses in children with nsASD applies to both males and females (45). Recent work has not demonstrated a correlation between autism severity and negative affect in toddlers with nsASD, and this relation has not been examined in NT or non-autistic toddlers with developmental delays, leaving an important avenue for future research (25). Some studies have investigated RSA as a predictor of ASD severity, but this work is also limited (36). In school-age children and older, there is mixed evidence for the relation between baseline RSA and autism severity in children with nsASD; however, no work has examined these relations in pre-school children.

Both negative affect and RSA have utility in predicting social anxiety as well. Negative affect has been shown to predict the onset of childhood anxiety throughout early development (47–50). Early social fear, a component of negative affect, has also been found to predict social anxiety symptoms later in childhood (51, 52). Because between 42 and 79% of autistic individuals have any co-occurring anxiety disorder, this component of the autism phenotype is an important avenue for future research (53). In the one study that examined the relationship between RSA and anxiety in nsASD, FXS, and NT individuals, Klusek et al. (40) did not find a significant association. Other work has shown a relationship between internalizing symptoms and RSA in nsASD (54). However, this work was done with school-aged children and adolescents, and little is known about these relations in young children.

An accumulating body of research utilizes biobehavioral measurement to predict autism features and social anxiety in FXS populations as well. Interestingly, despite the connection between early negative affect and autism symptoms in children with nsASD, no such relation seems to exist in children with FXS (27, 28). However, there is evidence to suggest that RSA in FXS increasingly diverges from NT RSA across early development (41), a trajectory that mirrors the onset of autism symptoms (42). Further, RSA at 24 months in NT males and those with FXS was found to predict autism features at 36 months, such that lower RSA was associated with more autism symptoms (41). In older boys with FXS, RSA dysfunction is associated with communicative difficulties common in autism, but not autism severity (40).

Negative affect has consistently predicted anxiety symptoms in children with FXS despite the lack of relation to autism symptoms, pointing to negative affect’s utility as a distinguishing diagnostic marker in this population. Infants and toddlers with FXS exhibit atypical longitudinal patterns of facial and behavioral social fear and these patterns are associated with withdrawal symptoms associated with social anxiety (55). Further, prospective longitudinal studies have shown that early negative affect predicts anxiety symptoms in males (28) and females (27) with FXS. In older children, RSA dysfunction is associated with pragmatic language deficits common in autism in older boys with nsASD, but not autism severity (40). Infants with FXS demonstrate a blunted RSA response to a social stressor in comparison to NT infants, suggesting that infant RSA may be an early marker of social anxiety in FXS (56). However, Hogan et al. (41) found that in NT males and those with FXS, RSA at 24 months was not predictive of anxiety symptoms at 36 months, underscoring the importance of taking development into account when investigating biobehavioral processes.

The challenge of quantifying social behavior in very young children is exacerbated when autism and social anxiety comorbidities are present in preschoolers with neurodevelopmental disorders and cognitive and language delays (8, 9). Therefore, studies that employ a multi-method approach including child observations and parent report while integrating biological markers hold great promise to advance our understanding of the independence and shared features across these multiple disorders. The present study addresses the following overarching research questions of whether baseline RSA or temperamental negative affect differ across groups and are associated with symptoms of autism and social anxiety within nsASD, FXS, and NT preschoolers. We hypothesized that (1) our nsASD and FXS would show higher levels of negative affect and lower levels of RSA than our NT groups, but would not differ from each other; and (2) RSA and negative affect would differentially predict autism and social anxiety between groups, such than negative affect would predict social anxiety, but not autism features, and RSA would predict autism.

## Materials and methods

### Participants

Participants were drawn from two ongoing longitudinal studies focused on early markers of autism and anxiety in nsASD and FXS across pre-school (National Institutes of Health R01MH90194, R01MH107573, and PI: Roberts). These studies adopted a multi-method biobehavioral approach with assessments focused on developmental level, heart activity, temperament, autism, and anxiety at 3, 4, and 5 years of age across three groups: nsASD, FXS, and NT. Participants were included if they had gestational age >37 weeks, English as the primary language in the home, and no other known medical conditions. Participants were recruited primarily from research and medical sites as well as social media sites specializing in nsASD or FXS. Participants enrolled in the NT group had no family history of autism, and they were confirmed not to have autism or developmental delay [i.e., Mullen Early Learning Composite (ELC) scores greater than 70] through study participation. Those in the FXS group were confirmed through a review of a genetic report of greater than 200 repeats of the CGG sequence on the *FMR*1 gene. Because our sample was recruited with existing FXS diagnoses by their healthcare providers and provided their own genetic reports, the DNA tests used varied across our sample, with most of the sample reporting Southern Blot Analysis and PCR. Participants in the nsASD group had an existing community diagnosis of autism. Autism symptom severity, diagnoses, and the presence of cognitive or developmental delays (i.e., Mullen ELC score less than or equal to 70 at the time of enrollment) for the FXS and nsASD groups were confirmed through study participation (greater detail below).

Given that the longitudinal parent study was ongoing, data for the current cross-sectional study were drawn from completed assessments with group matching and complete data prioritized. This resulted in a total of 120 male and female preschoolers across three groups including nsASD (*n* = 47, *n*_males_ = 40), FXS (*n* = 41, *n*_males_ = 29), or NT development (*n* = 32, *n*_males_ = 25). The three groups did not significantly differ in chronological age or sex distribution, and the nsASD and FXS groups did not differ in developmental quotient. Participant characteristics can be found in Table 1. Parents provided written informed consent before enrollment, and all procedures were approved by the Institutional Review Board at the University of South Carolina.

**TABLE 1:** Participant characterization.

	nsASD	FXS	NT
*n* (% male)	47 (85.1)	41 (70.7)	32 (78.1)
Age (months)	42.9 (5.5)	45.4 (5.7)	45.6 (5.7)
**Ethnicity**			
% Not Hispanic/Latino	95.7	95.1	96.9
% Hispanic/Latino	2.2	4.9	3.1
% Unknown	2.1	0	0
**Race**			
% White	78.7	65.9	87.5
% Black	10.6	4.9	6.3
% Other	2.1	4.9	0
% More than one race	6.4	24.4	6.2
**Clinical characterization means (SD)**			
Baseline RSA	5.64 (1.3)	5.82 (1.5)	6.04 (0.92)
Negative affect	3.96 (0.76)	3.74 (0.60)	3.50 (0.69)^a^
Social anxiety	2.58 (2.8)	3.03 (3.3)	1.91 (2.1)
ADOS-2 CSS	7.40 (1.7)	5.46 (2.7)	1.24 (1.2)^b^
Overall DQ	58.4 (13.5)	58.9 (14.9)	101.3 (16.2)^c^

RSA, respiratory sinus arrhythmia; DQ, developmental quotient based on Mullen Early Composite. DQ has a mean of 100. ADOS-2 CSS, autism diagnostic observation schedule calibrated severity score. CSS has a range of 1–10. ^a^nsASD group is significantly different from FXS and NT. ^b^All groups significantly differ from each other. ^c^NT group is significantly different from nsASD and FXS.

### Procedures

A combination of direct behavioral measurement and parent report was implemented to collect the data. The behavioral measures included developmental ability (DQ), baseline heart activity, and autism symptom severity. Parent-report measures of social anxiety and temperamental negative affect were completed using a paper and pencil format.

Autism diagnoses were assigned or ruled out through the larger longitudinal study using Clinical Best Estimate (CBE) procedures (22). A team that included a licensed psychologist who was an independent trainer for the ADOS-2 and at least two other team members who were research-reliable on the ADOS-2 reviewed cases using DSM-5 criteria for autism spectrum disorder (ASD) (5). Data reviewed during the CBE process included ADOS-2 scores and videos, clinical interviews, and behavioral ratings across the assessment, in addition to performance on cognitive and adaptive behavior assessments. An autism diagnosis was assigned only when children met DSM-5 criteria and symptoms were not better accounted for by another disorder (e.g., intellectual impairment).

### Measures

#### Baseline RSA

Baseline physiological regulation was indexed by mean RSA during a baseline video condition in which the child was seated and presented with an engaging, 5 min video without words. Heart activity was collected *via* electrocardiogram (ECG) during the baseline using an Actiwave Cardio Monitor (Alive Technologies; CamNtech Ltd., Cambridge, UK) at 1024 Hz. Heart rate was extracted using a threshold detection method in QRSTool software (57). A trained research assistant edited ECG data to correct false heart periods and artifacts using CardioEdit software, and data were only included if they met a threshold of 10% or fewer beats edited (58). CardioBatch was then used to sample sequential heart periods in 250 ms epochs and de-trend the data with a 21-point moving polynomial algorithm (59). Next, the data were filtered to remove variance associated with respiration rate (0.3–1.3 Hz), and RSA was estimated by transforming the variance to its natural logarithm. The mean RSA averaged across 30 s epochs during the baseline period was then taken as the independent variable of interest (57, 59).

#### Negative affect

Temperamental negative affect was assessed using the negative affect composite score from the Childhood Behavior Questionnaire (CBQ), a standardized measure of temperament from children aged 3 to 7 years (26). The negative affect composite includes items related to anger, frustration, fear, sadness, soothability, and the ability to recover from distress. For the scales comprising the Negative Affect Composite in 4- to 5-year-old children, internal consistency estimates (coefficient a) ranged from 0.66 to 0.80 (26). The factor structure of this temperament framework has been evaluated in populations with FXS, and it was largely retained for the negative affect composite (60). Negative affect is relatively stable in children with FXS across this timeframe, changing by less than half a point (27).

#### Autism symptoms

Autism symptom severity was measured using the Autism Diagnostic Observation Schedule, Second Edition [ADOS-2; (61)]. The ADOS-2 is a semi-structured play-based, semi-structured measure to elicit social interaction. Module 1, Module 2, or Module 3 of the ADOS-2 was administered by lab-reliable or research-reliable examiners, and module selection was determined by the child’s age and expressive language abilities. Calibrated severity scores (CSS) were utilized in the present study to account for symptom severity across modules and to provide a validated, continuous measure of autism symptomatology (62). Scores ranged from 1 to 10, with higher numbers reflecting more severe autism symptoms.

#### Social anxiety symptoms

Social anxiety was measured using the Preschool Anxiety Scale-Revised [PAS-R; (63)]. The PAS-R is a parent-report screening and diagnostic questionnaire for anxiety disorders in young children. In line with other studies (64), raw scores for the social anxiety scale were used in the present study because T-scores had a restricted range in the sample (*M* = 45, *SD* = 4.5). Although the PAS-R was not designed for use in populations with neurodevelopmental disabilities, it has been used in studies of children with autism, intellectual disabilities, FXS, and other genetic syndromes (65, 66). Social anxiety items have strong factor loadings on their hypothesized scale, and an adaptive version of the PAS-R shows strong internal consistency for this scale as well [0.74 < α < 0.77; (67, 68)].

#### Developmental ability

Developmental ability was measured with the Mullen Scales of Early Learning (MSEL), standardized assessments of early development for children from birth through 68 months (69). It evaluates development in the following five categories: Gross Motor, Visual Reception, Fine Motor, Receptive Language, and Expressive Language. ELC scores are derived from Visual Reception, Fine Motor, Receptive Language, and Expressive Language scores and utilized as a standardized metric of DQ.

### Analytic plan

All analyses were conducted in SPSS 26. A series of preliminary analyses were run including descriptive analyses for all variables of interest. Group differences in DQ and biological sex were also evaluated, and correlations between all study variables were conducted for all groups individually. We also evaluated whether baseline RSA or temperamental negative affect differed by conducting a one-way analysis of covariance (ANCOVA) to test for group and sex differences in RSA, with group and sex as the factor and RSA as the dependent variable. In line with prior work, DQ was included in the model as a covariate in RSA models due to its potential effect on the dependent variable (70). Finally, a one-way analysis of variance (ANOVA) was used to test for group and sex differences in negative affect, with group and sex as factors and negative affect composite score as the dependent variable. DQ was not included as a covariate for this analysis, because we have shown that it is an independent construct from temperament (27). These preliminary analyses are critical to confirm group differences that could contribute to the primary research question analyses and interpretation.

To address the primary research question of whether baseline RSA or temperamental negative affect are uniquely associated with symptoms of autism and social anxiety within nsASD, FXS, and NT groups, regression analyses were run. For each group, autism and social anxiety symptoms were regressed on RSA and negative affect scores, with DQ and sex included in all models as covariates.

## Results

Preliminary analyses indicated that the groups differed in DQ [*F*_(2, 117)_ = 99.2, *p* < 0.001] and ADOS-2 CSS [*F*_(2, 120)_ = 76.4, *p* < 0.001], but not Social Anxiety [*F*_(2, 110)_ = 1.35, *p* = 0.264]. *Post hoc* analyses demonstrated that the NT group had higher DQ than both clinical groups (*p*s < 0.001), but the nsASD and the FXS groups did not differ from each other (*p* = 0.884). In terms of ADOS-2 CSS scores, all groups differed from each other (*p*s < 0.001) with the nsASD group being the highest, FXS intermediate, and the NT group lowest. A Kruskal-Wallace test indicated that groups did not significantly differ in the distribution of sex (*h*^2^ = 2.64, *p* = 0.266). Means and standard deviations for all independent and dependent variables are shown in Table 1. Exploratory Pearson correlations among all key study variables for each group were conducted to understand the relation between RSA, negative affect, social anxiety, and autism severity before conducting the main study analyses. Baseline RSA and autism severity were significantly negatively correlated in both the nsASD group (*r* = −0.424, *p* = 0.008) and the FXS group (*r* = −0.364, *p* = 0.037). For the TD group, no correlations were significant.

**FIGURE 1:**
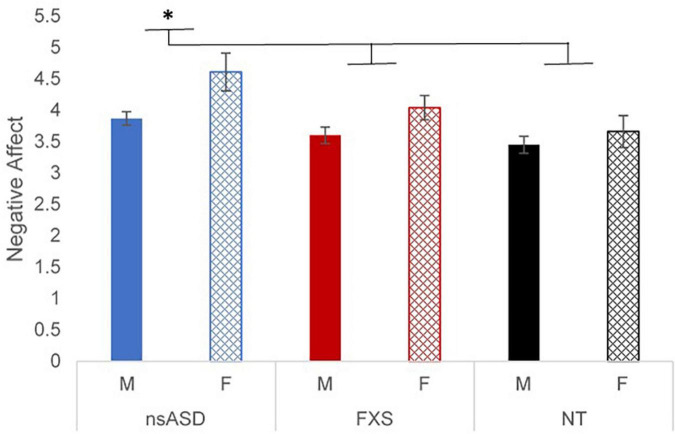
Estimated marginal means of negative affect ratings by group and sex. *p < 0.05 and ^**^p < 0.01.

To address whether there were group differences in RSA or negative affect, two univariate analyses were run. First, results of a univariate ANCOVA with RSA as the dependent variable, group as the factor, controlling for DQ, and indicated no significant effects by group [*F*_(2, 114)_ = 0.110, *p* = 0.896; partial *h*^2^ = 0.002], sex [*F*_(1, 114)_ = 0.117, *p* = 0.734; partial *h*^2^ = 0.001], or a group by sex interaction [*F*_(2, 114)_ = 0.766, *p* = 0.467; partial *h*^2^ = 0.014]. Next, a univariate ANOVA with negative affect as the dependent variable and group as the factor indicated that there were significant main effects for group [*F*_(2, 114)_ = 5.12, *p* = 0.007; partial *h*^2^ = 0.087] and sex [*F*_(1, 114)_ = 7.93, *p* = 0.006; partial *h*^2^ = 0.068], but no group by sex interaction [*F*_(2, 114)_ = 0.766, *p* = 0.467; partial *h*^2^ = 0.014]. *Post hoc* analyses suggested that the nsASD group had significantly higher negative affect than the FXS group (*p* = 0.038) and the NT group (*p* = 0.002); the FXS and NT groups did not differ (*p* = 0.148). In terms of sex effects, females had higher negative affect than their male peers (*p* = 0.006). A plot of the estimated marginal means by group and sex is presented in Figure 1.

**FIGURE 2:**
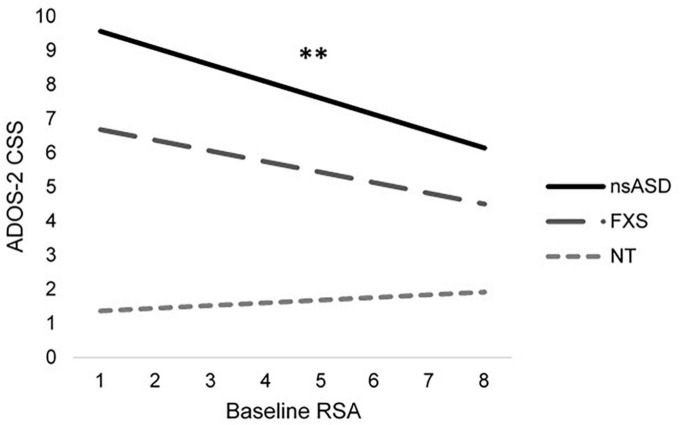
Modeled relationship between RSA and ASD severity for all groups. *p < 0.05 and ^**^p < 0.01.

To address the primary research question of whether baseline RSA or temperamental negative affect were associated with symptoms of autism and social anxiety across groups, individual models were run with DQ and sex included as covariates. Results are shown in Table 2. Analyses predicting ADOS-2 CSS from RSA and negative affect were significant for the nsASD [*F*_(4, 30)_ = 4.04, *p* = 0.010; Adjusted *R*^2^ = 0.264] and FXS groups [*F*_(4, 25)_ = 8.10, *p* < 0.001; Adjusted *R*^2^ = 0.495], but not for the NT group [*F*_(4, 26)_ = 0.616, *p* = 0.655; Adjusted *R*^2^ = −0.054]. In examining the individual predictors, RSA was significant for the nsASD group only, whereby a unit increase in RSA (i.e., more typical regulation) was associated with a 0.489-point decrease in CSS score (*p* = 0.008). Mullen ELC scores were also significantly related to autism severity in both the FXS and nsASD groups (*p*s < 0.05). Figure 2 illustrates the modeled relationship between RSA and autism severity for all three groups.

**TABLE 2:** Summary of linear regressions predicting social anxiety and ASD.

	Social anxiety			Autism severity		
	B (SE)	*t*	Adj. *R*^2^	B (SE)	*t*	Adj. *R*^2^
nsASD			0.074^+^			0.264*
Intercept	−7.24 (3.87)	−1.87		13.82 (1.75)	7.28**	
RSA	−0.064 (0.374)	−0.170		−0.489 (0.171)	−2.86**	
NA	1.46 (0.695)	2.10*		−0.317 (0.309)	−1.03	
DQ	0.076 (0.039)	1.93		−0.043 (0.018)	−2.39*	
Male	−0.604 (1.45)	−0.416		0.479 (0.667)	0.718	
FXS			0.248*			0.495**
Intercept	−11.9 (4.90)	2.90**		11.7 (3.12)	3.75**	
RSA	0.128 (0.446)	0.287		−0.312 (0.283)	−1.10	
NA	2.96 (1.04)	2.83**		0.695 (0.662)	1.05	
DQ	0.062 (0.043)	1.45		−0.124 (0.027)	−4.53**	
Male	−1.95 (1.38)	−1.42		0.410 (0.875)	0.469	
NT			−0.014			−0.054
Intercept	0.345 (3.51)	0.098		−1.17 (2.12)	−0.553	
RSA	0.290 (0.448)	0.648		0.079 (0.270)	0.293	
NA	0.773 (0.604)	1.28		0.210 (0.364)	0.576	
DQ	−0.028 (0.023)	−1.15		0.017 (0.015)	1.15	
Male	0.006 (0.941)	0.001*		0.062 (0.568)	0.110	

RSA, respiratory sinus arrhythmia; NA, negative affect; DQ, developmental ability. *p < 0.05. **p < 0.01. ^+^*p* < 0.10.

**FIGURE 3:**
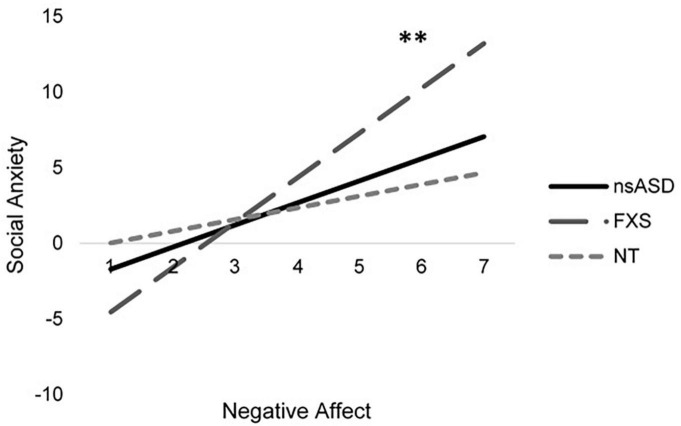
Modeled relationship between negative affect and social anxiety for all groups. *p < 0.05 and ^**^p < 0.01.

Analyses predicting social anxiety from RSA and negative affect indicate significant effects for the FXS group [*F*_(4, 25)_ = 8.10, *p* < 0.001; Adjusted *R*^2^ = 0.495] with a trend implicated for the nsASD group [*F*_(4, 38)_ = 2.39, *p* = 0.074; Adjusted *R*^2^ = 0.148]. Results were not significant for the NT group [*F*_(4, 26)_ = 0.897, *p* = 0.480; Adjusted *R*^2^ = −0.014]. However, these findings seem to largely be driven by the effect of negative affect, as RSA was not a significant individual predictor. In FXS, a unit increase in the negative affect score resulted in a 2.98-point increase in the social anxiety score (*p* = 0.009). In nsASD, a unit increase in the negative affect score resulted in a 1.46-point increase in the social anxiety score (*p* = 0.045). Figure 3 illustrates the modeled relationship between negative affect and social anxiety for all three groups.

## Discussion

The present study advances our understanding of biobehavioral underpinnings of social anxiety and autism symptoms in young children with nsASD and FXS with cognitive or developmental delays. Given the high prevalence and significant impairment of social anxiety that overlaps with autism features in both nsASD and FXS, this work is critical to identify discrete targets and timing for intervention. However, this work is also challenging given difficulties measuring symptoms in young children, especially when features of social anxiety may be less clear and when symptoms of autism and anxiety overlap. These challenges are magnified when studying young children with nsASD and FXS who present with cognitive and communication impairments that preclude the use of self-report measures and for whom the presentation of social anxiety may differ from NT controls. As such, implementing a biobehavioral approach is critical. Such approaches utilize objective markers that are detectable early in development and identify potential causes, rather than a sole focus on symptom presentation. In this study, we employed temperamental measures of negative affect that reflect biologically based indices of reactivity to environmental and social challenges complemented with physiological indices of RSA that, together, capture biological competence to manage challenges (23, 32). Negative affect and RSA have been linked to autism symptoms and social anxiety in both clinical and NT populations (25, 27, 36, 41, 47); however, the integration of these relationships has not been examined in young children with nsASD or contrasted to FXS. The present study addressed this gap in the field, and our findings indicate important patterns that are unique across clinical groups.

This study is one of a handful to contrast RSA in nsASD to FXS, and it is the first to do so in preschoolers including females, or to focus on participants with cognitive and language impairments which is often under-studied in nsASD (9, 71). Our findings indicate that baseline RSA was similar across the nsASD, FXS, and NT groups. This is consistent with some work that reports no differences across baseline RSA in nsASD (37, 40) or FXS (40, 56). However, our results contrast with studies that have reported lower RSA in nsASD (38, 39, 72). Likewise, the bulk of evidence from our lab and others has shown that RSA is lower in FXS (41–43, 73). Differences between the current study and those that report lower RSA in nsASD or FXS could be due to participant differences as we included females and focused on those with cognitive delays in the current study. This hypothesis is partially supported as there was no evidence of sex effects in either the nsASD or FXS groups; however, lower DQ was associated with reduced RSA in both neurodevelopmental groups but not in the NT controls. Thus, developmental level may be particularly important in understanding RSA for populations with cognitive delays. Given the fact that DQ intersects strongly with sex in FXS, with females typically less impaired, more work including a large number of females is needed to understand how sex and developmental level may influence baseline RSA in FXS.

Interestingly, we found that lower RSA predicted elevated autism symptom severity in the nsASD group only. Of note, this is the first study in preschoolers with nsASD that compares RSA to autism symptoms measured by the ADOS-2, a highly validated tool for measuring autism severity, though other work has addressed this question using parent-report measures of autism and social functioning (30, 72, 74). Given the theorized relationship between RSA and social engagement, this result suggests a mechanism through which underlying levels of arousal relate to greater social impairments in observed contexts (32). Our findings underscore the importance of taking both individual differences, as well as developmental stage, into account when considering candidates for biobehavioral markers.

The lack of a relationship between lower baseline RSA and elevated autism symptoms in the FXS group was unexpected and not consistent with previous studies (41, 42). However, recent findings indicate that the differences between young males with FXS and NT controls do not emerge until 29 months of age (41). Thus, the relationship of RSA to autism symptoms in FXS may strengthen across age, and our sample is young. Our approach to examining the effect of RSA to autism symptoms in a model that also includes negative affect which aligned with our research question may have influenced our results and reduced power to detect these relationships in isolation. The level of autism symptom severity is another important factor to consider as the nsASD group had elevated autism symptom severity, as expected, suggesting that the relationship between baseline RSA and autism symptom severity might be stronger in groups with more severe autism symptoms. Finally, the finding that lower DQ was associated with lower baseline RSA in the FXS group alone, despite the nsASD and FXS groups being matched on DQ, is important to note as it suggests that developmental level may be a more salient factor in determining physiological factors than in nsASD.

The present study did not reveal a relationship between baseline RSA and social anxiety in any group, a finding that aligns with prior literature in older children with nsASD (40) and infants with FXS (41, 56). Although there is evidence to suggest that RSA may be an important infant marker of social fear (47), our findings suggest it may not be as relevant later in development. Further, other work has suggested that general physiological arousal measured by other cardiac indices (e.g., heart rate) may be more predictive of anxiety in FXS (41). Similarly, the present study indicated that negative affect was not associated with autism symptoms in any group, corroborating findings from other studies in FXS (27, 28) and nsASD (25). Taken together, these findings support the notion that, although nsASD is associated with elevated negative affect, temperamental traits measure early emerging biologically based personality characteristics that are distinct from autism symptoms.

Our findings did reveal group differences in negative affect, whereby children with nsASD had greater negative affect than both the FXS and NT groups who did not differ from each other. These findings expand upon the existing literature on temperamental negative affect in neurodevelopmental disabilities. Our results replicate and extend prior work demonstrating that 26-month-old toddlers with nsASD have higher negative affect than their NT peers and non-autistic developmental delayed toddlers (25). Our finding that FXS and NT children have similar levels of negative affect is in line with other work demonstrating that preschoolers with FXS exhibit the same (27), or even lower (29), negative affect than NT controls. Of relevance, evidence suggests that negative affect in FXS increases across early childhood; thus, the trajectory of negative affect may not be linear highlighting the importance of age for this group.

Interestingly, research on temperament in nsASD has shown that temperamental regulation (i.e., effortful control or behavioral inhibition) is associated with autism severity in nsASD (25) and FXS (75). Given that these types of behavioral regulation have important developmental and biological underpinnings, these findings point to another potential category of regulatory dysfunction that is associated with autism. This literature suggests that for those with neurodevelopmental disorders, it may not be temperamental negativity, but rather the underlying regulation of emotions that is most indicative of symptom severity.

To our knowledge, no studies have contrasted negative affect in FXS to nsASD so our findings extend this literature to suggest that elevated negative affect also distinguishes pre-school children with nsASD from those with FXS. Taken together, these findings suggest that elevated negative affect may be representative of a nsASD -specific temperamental vulnerability that persists throughout development. In light of other research that indicates underlying social vulnerabilities distinguish nsASD from FXS (64), the present study furthers our understanding of biobehavioral differences between these disorders. Interestingly, we also found sex differences in negative affect across our sample with females exhibiting higher levels compared to their male peers. Previous work in children with FXS and NT children has shown sex differences in developmental trajectories of negative affect, with females and males expressing different levels at different points in time, pointing to the importance of continued longitudinal work in this area (27).

The present study found that negative affect significantly predicted anxiety symptoms in children with FXS and marginally predicted anxiety in nsASD (27, 28). This is in line with work documenting biobehavioral similarities in anxiety among those with FXS and nsASD (76–78). Negative affect is a key theoretical mechanism of the development of anxiety in NT pediatric populations (5, 79); our findings provide evidence that this mechanism is preserved in children with neurodevelopmental disorders, particularly for FXS, which is characterized by elevated anxiety (80). The absence of this relation in our NT group may be due to very low levels of social anxiety in our sample.

### Limitations and future directions

The present study benefited from a relatively large, well-matched, and controlled sample of children across all groups of interest that allowed us to test for unique biobehavioral mechanisms at specific developmental periods. However, this work was cross-sectional which precludes examination of the progression of symptoms across ages in these neurodevelopmental disorders. Prior research has examined biological and physiological underpinnings of anxiety and autism in FXS (27, 41), and future work would do well to consider how developmental trajectories impact the onset of symptoms in young individuals with other neurodevelopmental disorders, including nsASD. In addition, due to power limitations, our FXS sample comprised children with and without concomitant autism diagnoses. It is yet unknown whether a relation between negative affect and later autism symptoms also exists in children with FXS who have a stable autism diagnosis, and this question offers an important avenue for future research. Finally, we did not have specific data regarding *FMR1* repeats, mosaicism, or methylation status for our FXS. Given that this information is emerging as another useful marker of neurobehavioral profiles in this population, future work should continue to investigate molecular profiles of social behavior as well, as they have been shown to stratify groups based on neurobehavioral functioning and to track treatment response in clinical trials (16, 81).

In sum, this work confirms and extends the existing literature in several important ways. First, it suggests that between 3 and 4 years of age, children with nsASD differ from their NT peers with elevated negative affect, but children with FXS do not. Second, it verifies prior work in FXS indicating that negative affect predicts social anxiety symptoms but not autism severity; it also extends this finding to a cohort of children with nsASD and cognitive delays. Finally, our findings indicate that baseline RSA predicted autism severity for nsASD only, which is useful given the potential consequences of early regulatory atypicality on later symptoms in nsASD and FXS (25, 27, 42). The present study provides evidence for between-group as well as within-group heterogeneity in neurodevelopmental disorders, and future work should also consider how biobehavioral markers can help refine our understanding of individual disorders. Our findings can be used to further disentangle the ways that biological markers index the relation between the presence of underlying diagnoses with neural underpinnings and observed symptom severity.

## Data availability statement

The raw data supporting the conclusions of this article will be made available by the authors, without undue reservation.

## Ethics statement

The studies involving human participants were reviewed and approved by the University of South Carolina. Written informed consent to participate in this study was provided by the participants’ legal guardian/next of kin.

## Author contributions

CW and JR designed the study. CW conducted the analysis and interpretation of the data, with contributions from JR. CW drafted the manuscript. JR was extensively involved in revisions. Both authors have given final approval for the version to be published.

## Footnotes

1 In line with journal use guidelines informed by autistic self-advocates and stakeholders in the autism community, we refer to ASD as autism throughout this manuscript except when we use the abbreviation to delineate our groups (1, 2). Similarly, we have decided to use identity-first language as much as possible (3, 4).
